# Supplementary material for: Cardiovascular diseases worsen the maternal prognosis of COVID-19
Source: PLoS One. 2023 Feb 7;18(2):e0266792. doi: 10.1371/journal.pone.0266792 (PMC9904457; doi:10.1371/journal.pone.0266792)
Supplement: Attachment — Submitted filename: ResponsestoReviewers.pdf [file pone.0266792.s001.pdf]

Prof. Vipa Thanachartwet, Academic Editor
PLOS ONE

Dear Prof. Thanachartwet,

Attached please find our revised paper [PONE-D-22-08926], “Cardiovascular diseases worsen the maternal prognosis of COVID-19”. We also attach a reply to the Referee’s comment. We sincerely appreciate the comments of the review team. We believe the current revision addresses all the comments on the previous submission.

Thank you in advance for considering this revision.

Best regards,

Luciana Graziela de Godoi

Response to the comments of the Editor

We would like to thank you for your comments. In what follows, we state each of the comments in bold, and describe our response in roman.

1. Please ensure that your manuscript meets PLOS ONE's style requirements, including those for file naming.

Response: Thank you for the comment. The manuscript as well as the file naming have been revised according to the PLOS ONE style requirements.

2. Thank you for stating the following in the Funding Section of your manuscript:

"This research was funded by Bill & Melinda Gates Foundation, National Council for Scientific and Technological Development (CNPQ - Brasília, Brazil), and Foundation for Research and Innovation Support of the State of Espírito Santo (FAPES - Espírito Santo, Brazil) are funding source."

We note that you have provided funding information. However, funding information should not appear in the Funding section or other areas of your manuscript. We will only publish funding information present in the Funding Statement section of the online submission form.

Please remove any funding-related text from the manuscript and let us know how you would like to update your Funding Statement. Currently, your Funding Statement reads as follows:

"This work was supported, in whole or in part, by the Bill & Melinda Gates Foundation (INV-027961). Under the grant conditions of the Foundation, a Creative Commons Attribution 4.0 Generic License has already been assigned to the Author Accepted Manuscript version that might arise from this submission. This work is also funded by CNPq (Award Number: 445881/2020-8) and FAPES (Award Number: 007/2021)."

Please include your amended statements within your cover letter; we will change the online submission form on your behalf.

Response: Thanks. We deleted the funding-related text from the manuscript and inserted it in the cover letter.

The following paragraph was excluded:

> *"Funding: This research was funded by Bill & Melinda Gates Foundation, National Council for Scientific and Technological Development (CNPQ - Brasília, Brazil), and Foundation for Research and Innovation Support of the State of Espírito Santo (FAPES - Espírito Santo, Brazil) are funding source."*

3. We note that you have stated that you will provide repository information for your data at acceptance. Should your manuscript be accepted for publication, we will hold it until you provide the relevant accession numbers or DOIs necessary to access your data. If you wish to make changes to your Data Availability statement, please describe these changes in your cover letter and we will update your Data Availability statement to reflect the information you provide.

Response: Thanks. We updated the data access link in the manuscript and cover letter.

Previous paragraph:

> “***Data Availability Statement:*** *Datasets are available in the Kaggle public repository at the URL: https://www.kaggle.com/datasets/agatharodrigues/papermaternal. These data were derived from the following resources available in the public domains: https://opendatasus.saude.gov.br/dataset/bd-srag-2020 and https://opendatasus.saude.gov.br/dataset/bd-srag-2021, obtained on May 5, 2021. All the R code and documentation are available in the GitHub repository at https://github.com/observatorioobstetrico/COVID19_CVD.*”

Modified paragraph:

> “***Data Availability Statement:*** *Datasets are available in the Kaggle public repository at the URL: https://www.kaggle.com/datasets/agatharodrigues/papermaternal. These data were obtained on May 5, 2021 from the following public domain available by the DATASUS: https://opendatasus.saude.gov.br/dataset. All the R code and documentation are available in the GitHub repository at https://github.com/observatorioobstetrico/COVID19_CVD.*”

4. Your ethics statement should only appear in the Methods section of your manuscript. If your ethics statement is written in any section besides the Methods, please delete it from any other section.

Response: Thanks. We have kept the ethical statement only in the "Methods" section. The following paragraphs were excluded:

> *“Institutional Review Board Statement: Ethical review and approval were waived for this study due to an open-base study, according to Brazilian regulations.*
>
> *Informed Consent Statement: Patient consent was waived due to it is a populational study in an open anonymized database, according to Brazilian regulations.”*

Response to the comments of the Reviewer 1

This is an observational study with a large sample size on the evaluation whether the presence of CVD in pregnant women and in postpartum women with COVID-19 is associated with a worse prognosis. Authors used an anonymized open database. They concluded that "Hospitalized obstetric patients with CVD and COVID-19 are more symptomatic." and that "Their management demand more ICU admission and ventilatory support and the mortality is higher."

I think that this is a publishable work, but I have some points that need reconsideration.

Response: Thank you for your kind comments. In what follows, we tried to state each of the comments in bold, and describe our response in roman. Phrases or words added to the revised manuscript are highlighted in yellow color.

1. In the abstract, as in the whole text, there must be clearer distinctions for the groups studied and compared.

Response:

Thanks. We included in the abstract and also in the methods and results the division of groups to make the comparison between them clear.

> "*The patients were divided into two groups according to the presence or absence of CVD (CVD and non-CVD groups)*"

2. In the introduction section, the rationale should be revisited. The aim of the study should be clearly stated in the last sentence.

Response: Thanks. We revised the introduction which was rewritten for clarity.

Current text modified:

> ***"1. Introduction***
>
> *Maternal mortality is one of the most sensitive markers of health care because it has multiple economic and social impacts. Brazil has a maternal mortality rate of 59.1/100,000 live births (1), above the values considered acceptable by the World*

Health Organization, which established as a millennium goal for Brazil rates below 35/100,000 live births (2).

The presence of cardiovascular diseases (CVD) is among the main indirect causes of maternal mortality in the world, mainly in developed countries (3–5). In Brazil, CVD accounted for 7% to 9.6% of maternal deaths in the years 2011 to 2019 (6).

During the COVID-19 pandemic, maternal mortality has increased significantly in the world. Currently, the postpartum period has been recognized as a risk factor for complications of this infection (7). By the time this article was written, Brazil had recorded 13,769 cases of Severe Acute Respiratory Syndrome (SARS) and 1,461 deaths (12.3%) due to COVID-19 during pregnancy and the postpartum period (8).

Since the Influenza A (H1N1) pandemic in 2009, the Brazilian Ministry of Health has since established and maintained the Influenza Epidemiological Surveillance Information System (Sistema de Vigilância Epidemiológica de Gripe, SIVEP-Gripe), with the possibility of access to data without the individual identification of the patient (anonymized data), on an open basis (9,10).

During the COVID-19 pandemic, the notification in the SIVEP-Gripe of cases of patients with SARS hospitalized in all health units in the country has been compulsory. It has enabled the evaluation of diagnosis, demographic and epidemiological data, and the evolution of cases. The case definition of SARS is of an individual with flu-like syndrome with at least two of the following signs and symptoms: fever, chills, sore throat, headache, cough, rhinorrhea, olfactory or taste disorders. They often present with dyspnea/respiratory distress, persistent chest pressure, O2 saturation less than 95% in ambient air, or cyanosis of the lips or face (11).

Studies reported that CVD is an independent risk factor for severe cases of COVID-19 in the general population (12,13). However, there are no studies that have evaluated whether the presence of CVD in pregnant and postpartum women with COVID-19 can be considered a risk factor for worse disease evolution, including a higher prevalence of symptoms and a higher risk of ICU admission, orotracheal intubation and death."

3. In the materials and methods, parts from there should be moved in the introduction section; authors should make clear what their comparisons should be and the exact methods; this should be followed in all the next sections, for the reader to understand exactly the correlations. The hospitalized COVID positive patients could be divided according to trimesters (as the results show afterwards), to their state (for e.g. incubated or not e.t.c), days of hospitalization, of NICU stay etc.; other comorbidities should be discriminated – for example a subgroup analysis there might be of essence.

Response: Thanks. We agree. The following paragraphs have been moved to the introduction.

"*Since the Influenza A (H1N1) pandemic in 2009, the Brazilian Ministry of Health has since established and maintained the Influenza Epidemiological Surveillance*

Information System (Sistema de Vigilância Epidemiológica de Gripe, SIVEP-Gripe), with the possibility of access to data without the individual identification of the patient (anonymized data), on an open basis (9,10). Thus, according to Brazilian regulations, there is no need for prior approval by institutions board review.

During the COVID-19 pandemic, the notification in the SIVEP-Gripe of cases of patients with SARS hospitalized in all health units in the country has been compulsory. It has enabled the evaluation of diagnosis, demographic and epidemiological data, and the evolution of cases. The case definition of SARS is of an individual with flu-like syndrome with at least two of the following signs and symptoms: fever, chills, sore throat, headache, cough, rhinorrhea, olfactory or taste disorders. They often present with dyspnea/respiratory distress, persistent chest pressure, O2 saturation less than 95% in ambient air, or cyanosis of the lips or face (11).”

We tried to define more clearly the groups being compared. For this, we rewrite the phrase adding comments.

“We selected cases of SARS with a confirmed diagnosis by the Ministry of Health of COVID-19 who had been hospitalized. Confirmed cases were those with positivity in laboratory tests (PCR, antigen test or serology), those with a highly suspicious image, and those with a compatible clinic in a patient in contact with a positive case (clinical-epidemiological confirmation) at the time when tests were scarce. After that, we selected pregnant and postpartum women aged 10 to 55 years old with data regarding their CVD status. In the SIVEP GRIPE data dictionary, CVD is defined as “presence of cardiovascular disease”, excluding those of neurological and nephrological causes that are pointed out in another field. The patients were divided into two groups according to the presence or absence of CVD (CVD and non-CVD groups).”

4. The results should contain the exact results of the comparisons.

Response: Thanks. In Figure 2, we enter the absolute data. In all tables, we reviewed the denomination of the groups as described in the abstract and in the methods.

Figure 2. *Absolute frequency and percentage of monthly growth rate of COVID-19 cases according to the month of the first symptom in the CVD and non-CVD groups.*

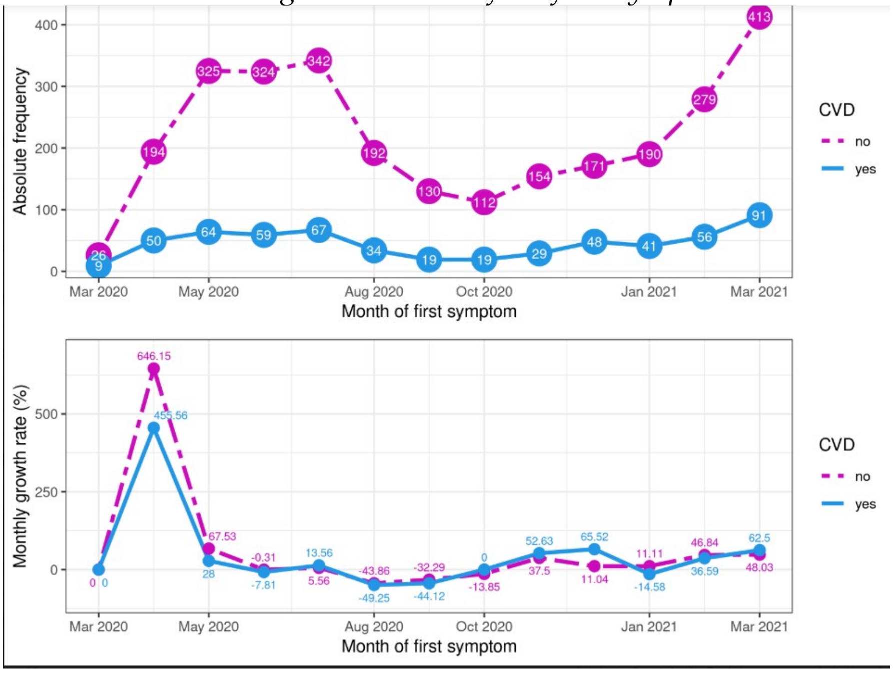

Table 1. *Demographic and clinical characteristics of pregnant and puerperium women hospitalized with COVID-19 confirmed according to the presence or absence of Cardiovascular Disease - Brazil, 02/16/2020 to 04/17/2021.*

Feature	CVD group	Non-CVD group	*p*
**AGE (YEARS)**			
Average ± SD	34.76 ± 8.52	30.00 ± 7.43	<0.001
**AGE GROUP (YEARS)**	**N (%)**	**N (%)**	
<20	10 (1.7)	237 (8.0)	<0.001
20-34	292 (48.5)	1895 (64.0)	
≥ 35	300 (49.8)	828 (28.0)	
**ETHNIC GROUP**	**N (%)**	**N (%)**	
Asian	2 (0.4)	24 (0.9)	0.007
White	214 (42.2)	975 (37.9)	
Indigenous	1 (0.2)	30 (1.2)	
Mixed	244 (48.1)	1375 (53.5)	
Black	46 (9.1)	167 (6.5)	
**EDUCATION**	**N (%)**	**N (%)**	
No schooling	2 (0.7)	11 (0.7)	0.241
1st to 5th grade	33 (12.2)	123 (7.9)	
6th to 9th grade	52 (19.2)	307 (19.7)	
High school	140 (51.7)	857 (55.0)	
College	44 (16.2)	260 (16.7)	
**MOMENT OF PREGNANCY**	**N (%)**	**N (%)**	
1st trimester	34 (5.6)	161 (5.4)	<0.001
2nd trimester	120 (19.9)	471 (15.9)	
3rd trimester	248 (41.2)	1199 (40.5)	
Gestational age ignored	30 (5.0)	84 (2.8)	
Puerperium	170 (28.2)	1045 (35.3)	
**RISK FACTORS AND COMORBIDITIES**	**N (%)**	**N (%)**	
Hemopathy (n=3387)	9 / 437 (2.1)	31 / 2950 (1.1)	0.113

Hepatopathy (n= 3364)	5 / 431 (1.2)	12 / 2933 (0.4)	0.056
Asthma (n= 3369)	40 / 436 (9.2)	199 / 2933 (6.8)	0.087
Diabetes (n= 3417)	158 / 470 (33.6)	337/2947 (11.4)	<0.001
Neuropathy (n=3374)	9 / 431 (2.1)	28 / 2943 (1.0)	0.062
Pneumopathy (n=3373)	13 / 437 (3.0)	31 / 2936 (1.1)	0.002
Immunosuppression (n=3365)	15/ 433 (3.5)	58 / 2932 (2.0)	0.071
Nephropathy (n=3359)	19/ 433 (4.4)	36 / 2926 (1.2)	<0.001
Obesity (n= 3355)	105 / 449 (23.4)	255 / 2906 (8.8)	<0.001
**NUMBER OF COMORBIDITIES (n=3460)**	**N (%)**	**N (%)**	
0	234 (46.8)	2126 (71.8)	<0.001
1	186 (37.2)	701 (23.7)	
2	64 (12.8)	116 (3.9)	
≥ 3	16 (3.2)	17 (0.6)	

5. The limitations of the study should be presented.

Response: The limitations of the study are pointed out in the "discussion" section.

> *"Among the limitations of the study, we should consider that the completion of the notification system included information about the presence or absence of CVD in 39.58% of the notifications, with a high percentage of loss. The difficulty in population-based use was also portrayed by other researchers. In the USA, for instance, only 5.8% (7162) of 122653 cases reported to the CDC had information about health conditions (33). In addition, we found limitation on the type of CVD, since the notification form does not allow etiological distinction and interpretation of access to ICU admission in Brazil. This is because only 15% of maternity hospitals in our country have adult ICU beds (34). Another weakness of our study is the lack of information on the evolution of CVD before COVID-19 infection, including treatments used, duration of illness and quality of follow-up."*

6. The conclusions should adhere to the exact aim, findings and type of the study presented.

Response: Thanks. As per your suggestion, we have rewritten the introduction making the objectives clearer, which are now completely compatible with the conclusions.

> *Introduction:*
>
> *"However, there are no studies that have evaluated whether the presence of CVD in pregnant and postpartum women with COVID-19 can be considered a risk factor for worse disease evolution, including a higher prevalence of symptoms and a higher risk of ICU admission, orotracheal intubation and death."*

Conclusion:

“Obstetric patients with CVD and COVID-19 who are hospitalized are more symptomatic. They require ICU hospitalization and ventilatory support. Moreover, they have a higher mortality rate than COVID-19 patients without CVD. Mortality in this group may be a consequence of the association between the severity of COVID-19 infection in pregnant women associated with the presence of CVD (which are, by themselves, are recognized risk factor for ICU admission, orotracheal intubation, and death during pregnancy and puerperium). Further studies are needed in this group of patients to determine the main associated risk factors, analyze individually the etiology of CVD, and thus allow improvements in care and consequent reduction in maternal mortality rates.”

Response to the comments of the Reviewer 2

The authors present convincing data on the worse prognosis of pregnant women with COVID-19 infection and cardiovascular diseases (CVD). However, some points need to be clarified.

Response: Thank you for your kind comments. In what follows, we tried to state each of the comments in bold, and describe our response in roman. Phrases or words added to the revised manuscript are highlighted in yellow color.

1. Could the authors present both groups of women with the WHO classification of symptoms (asymptomatic, mild-moderate, and severe infection).

Response: Thanks for your comment. We chose not to use the WHO classification, but to evaluate the symptoms individually since 574/596 (96.3%) of the cases with CVD and 2685/2941 (91.3%) of the cases in the non-CVD group had at least one symptom. The small percentage of patients who did not show symptoms of COVID-19 infection may represent pregnant and postpartum women who were hospitalized for other reasons but who tested positive at the time of admission. Our objective in this comparison was especially to assess whether respiratory symptoms were more frequent in patients with CVD, which was observed by us in clinical practice and also in this study where 83.7% of women in the CVD group had at least one respiratory symptom while in the non-CVD group this occurred in 68.1% of cases. Another relevant fact is that SIVEP influenza only presents data on hospitalized cases or that died at home, and in Brazil there is another database with mild and asymptomatic cases, but due to the anonymization of the data it is not possible to make the necessary linkage to avoid duplication of patients.

2. Lack of information on the baseline and follow-up of CVD patients. Please provide how many years CVD before COVID-19 infection, treatment for CVD (yes or no) and

type of treatment, lost of follow-up during COVID-19 infection, treatment delay during COVID-19 infection.

Response: Thanks for your comment. As it is a national flu syndrome case reporting system, details on the diseases that could certainly contribute to this study are not available. We include this weakness in the discussion.

> *"Another weakness of our study is the lack of information on the evolution of CVD before COVID-19 infection, including treatments used, duration of illness and quality of follow-up."*

3. Please explain why the propensity score matching was used as the method for balancing weights.

Response: In order to understand the need for considering the propensity score matching in the study, it is necessary to know the differences between experimental and observational studies.

The objective of randomization in experimental studies is to obtain groups of subjects that are, at baseline and on average, identical in terms of observed and unobserved characteristics. Therefore, any average difference between the groups after applying different treatments can only be attributed to the treatment effects. In observational studies, by definition, there is no prior randomization and the group that received the treatment can be substantially different from the group that did not receive or received another type of treatment (controls). For instance, if the distribution of age is different between the treatment and control groups, the age variable is a potential confounding variable in the study and it can lead to biased treatment effect estimates. This difference between the groups is also known in the literature as sample selection bias. Other common sources of bias in observational studies are gender, ethnicity, comorbidities, etc. Particularly, our study is observational and we consider age, ethnic group, obesity, and the presence of diabetes as possible confounding variables between the groups CVD and non-CVD. Propensity scoring, proposed by Rosenbaum & Rubin (1983), is a set of methodologies that aim to reduce sample selection bias in observational studies. For this purpose, propensity score methods offer a way to balance groups by matching treatment and control units based on a set of covariates that, in general, are considered the potential confounding variables. Rosenbaum & Rubin (1983) have demonstrated that subjects in treated and control groups with equal propensity score have the

same distributions of the observed covariates and, by consequence, the treated and control groups now have similar characteristics with respect to the possible confounding variables.

Rosenbaum P.R., Rubin D.B. The central role of the propensity score in observational studies for causal effects. Biometrika. 1983;70:41–55.

Response to the comments of the Reviewer 3

The paper has major problems, denying it the right to be published.

Response: Thank you for your comments. In what follows, we tried to state each of the comments in bold, and describe our response in roman. Phrases or words added to the revised manuscript are highlighted in yellow color.

1. The main problems are

(1) The large proportion of missing data of the patients' background diseases, which can highly influence the results.

Response: We agree that there is a large proportion of missing data, which was even pointed out as one of the weaknesses of the study, however the loss of data in this study was lower than in other similar studies such as the one by the CDC where only 5.8% (7162) of 122653 cases reported had information about health conditions.

It is worth mentioning that we only use cases with the information referred to as "presence" or "absence" of the disease, that is, we do not use it if the field has not been filled in. With this, we hope to minimize the bias of the study.

(2) The lack of information on CVD definition.

Response: Thanks for your comment. As it is a database of the notification system, we only have the information present in the form. In the SIVEP GRIPE data dictionary, this data is defined as "presence of chronic cardiovascular disease", excluding those of neurological and

nephrological causes that are pointed out in another field. We chose to insert this information in the abstract and in the methods.

> *"In the SIVEP GRIPE data dictionary, CVD is defined as "presence of cardiovascular disease", excluding those of neurological and nephrological causes that are pointed out in another field."*

(3) **The diagnosis method of COVID-19, which is clinically based and not confirmed by PCR in my understanding.**

Response: Only cases of COVID-19 confirmed by the Ministry of Health were included. Confirmed cases were those with positivity in laboratory tests (PCR, antigen test or serology), those with a highly suspicious image, and those with a highly suspicious clinical in a patient in contact with a positive case (clinical-epidemiological confirmation) at the time when tests were scarce. Among all the cases included, 3392 (95.2%) presented a positive laboratory test, 126 (3.5%) were considered confirmed through imaging alone, and 44 (1.2%) were considered positive when clinically and epidemiologically confirmed.

We include this information more clearly in the methods and results.

> ***2. Materials and Methods***
>
> *"We selected cases of SARS with a confirmed diagnosis by the Ministry of Health of COVID-19 who had been hospitalized. Confirmed cases were those with positivity in laboratory tests (PCR, antigen test or serology), those with a highly suspicious image, and those with a compatible clinic in a patient in contact with a positive case (clinical-epidemiological confirmation) at the time when tests were scarce. After that, we selected pregnant and postpartum women aged 10 to 55 years old with data regarding their CVD status. In the SIVEP GRIPE data dictionary, CVD is defined as "presence of cardiovascular disease", excluding those of neurological and nephrological causes that are pointed out in another field. The patients were divided into two groups according to the presence or absence of CVD (CVD and non-CVD groups)."*
>
> ***3. Results***
>
> *"In the period between the 8th epidemiological week of 2020 and the 15th epidemiological week of 2021 in a consultation held on May 5, 2021, we found 1,876,953 reported cases of COVID-19. We selected only the confirmed and*

hospitalized cases of COVID-19 in pregnant and postpartum women aged 10 to 55 years who had in their notification the information about the presence or absence of CVD (Figure 1). Among all the cases included, 3392 (95.2%) presented a positive laboratory test, 126 (3.5%) were considered confirmed through imaging, and 44 (1.2%) were considered positive when clinically and epidemiologically confirmed."

2. English editing is required.

Response: Thank you for your comment. English has been revised.

3. Abstract: "cardiovascular diseases" definition required. Which diseases were included?

Response: Thank you for your comment. We accept your suggestion and insert the data dictionary definition in the abstract.

*"**Abstract:** Cardiovascular diseases (CVD) are a risk factor for severe cases of COVID-19. There are no studies evaluating whether the presence of CVD in pregnant and postpartum womenwith COVID-19 is associated with a worse prognosis. In an anonymized open database of the Ministry of Health, we selected cases of pregnant and postpartum women who were hospitalized due to COVID-19 infection and with data regarding their CVD status. In the SIVEP GRIPE data dictionary, CVD is defined as "presence of cardiovascular disease", excluding those of neurological and nephrological causes that are pointed out in another field. The patients were divided into two groups according to the presence or absence of CVD (CVD and non-CVD groups). Among the 1,876,953 reported cases, 3,562 confirmed cases of pregnant and postpartum women were included, of which 602 had CVD. Patients with CVD had an older age (p<0,001), a higher incidence of diabetes (p<0,001) and obesity (p<0,001), a higher frequency of systemic (p<0,001) and respiratory symptoms (p<0,001). CVD was a risk factor for ICU admission (p<0,001), ventilatory support (p=0.004) and orotracheal intubation in the third trimester (OR 1.30 CI95%1.04-1.62). The group CVD had a higher mortality (18.9% vs. 13.5%, p<0,001), with a 32% higher risk of death (OR 1.32 CI95%1.16-1.50). Moreover, the risk was increased in the second (OR 1.94 CI95%1.43-2.63) and third (OR 1.29 CI95%1.04-1.60) trimesters, as well as puerperium (OR 1.27 IC95%1.03-1.56). Hospitalized obstetric patients with CVD and COVID-19 are more symptomatic. Their management demand more ICU admission and ventilatory support and the mortality is higher."*

4. Methods

4.1. "The case definition of SARS is of an individual with flu-like syndrome"- A confirming PCR test was not obligatory for diagnosis?

Response: This is the case definition used by the Ministry of Health, defining the cases that are of compulsory notification. In the investigation form, there is the possibility of including the etiology and the test used for diagnostic confirmation. In Brazil, as in other countries, there were times when there was no COVID-19 test available. Confirmed cases were those with positivity in laboratory tests (PCR, antigen test or serology), those with a highly suspicious image, and those with a compatible clinic in a patient in contact with a positive case (clinical-epidemiological confirmation) at the time when tests were scarce. Among all the cases included, 3392 (95.2%) presented a positive laboratory test, 126 (3.5%) were considered confirmed through imaging, and 44 (1.2%) were considered positive when clinically and epidemiologically confirmed.

4.2. Children were included (age 10-18), in my opinion this population deserves a sub-analysis.

Response: Thanks for your comment. Brazil chose to include adolescents, that is, girls aged 10 and over, in the definition of women of childbearing age, due to the high prevalence of teenage pregnancy in our country. In our series, the number of patients in this age group was small (10 patients under 18 years of age, all in the non-CVD group, corresponding to 0.29% of our sample). In this work, we did not analyze this subgroup, however, we are working on the evaluation of cases of COVID-19 in pregnant and postpartum women aged 10 to 18 years.

4.3. "To control selection biases in non-experimental studies, the CVD and non-CVD groups were balanced with respect to.."- "balanced" needs to be written "adjusted".

Response: The term "balanced" is related to the main of the propensity score. Proposed by Rosenbaum & Rubin (1983), the propensity score is a balancing score because conditional on the propensity score, the distribution of observed baseline covariates will be similar between treated and untreated subjects. Please, read the answer given to the third question of the second reviewer about the propensity score matching.

Rosenbaum P.R., Rubin D.B. The central role of the propensity score in observational studies for causal effects. Biometrika. 1983;70:41–55.

5. Results

5.1. "We selected only the confirmed and hospitalized cases of COVID-19"- The confirmation methods needs to be specified in details in the "Methods" section.

Response: Thanks for your comment. We accept your suggestion and enter the details in the methods section.

> "We selected cases of SARS with a confirmed diagnosis by the Ministry of Health of COVID-19 who had been hospitalized. Confirmed cases were those with positivity in laboratory tests (PCR, antigen test or serology), those with a highly suspicious image, and those with a compatible clinic in a patient in contact with a positive case (clinical-epidemiological confirmation) at the time when tests were scarce. "

5.2. 602 of 3,562 cases had CVD? It is a young population, so it seems to me to be over-estimated. Please, again, specify the definition of CVD in this study. Which ICD codes were included?

Response: Thanks for your comment. As it is an anonymized database extracted from the compulsory notification forms, we used the available data, which would be the “presence” or “absence” of cardiovascular disease. According to the Ministry of Health's data dictionary, CVD was defined as "chronic cardiovascular disease". There is no information about the ICD codes available.

The frequency of CVD in our study was comparable to that reported in the North American population study with 8,207 pregnant women with a positive laboratory test for COVID-19, in which 14% of the pregnant women had CVD. Although the population studied was young, we only evaluated hospitalized patients, which contributes to the higher incidence of previous chronic diseases.

5.3. “We noticed that the absolute numbers were increased in both groups from March to May 2020, with a high level until July.” – What absolute numbers?

Response: Thanks for your comment. The absolute numbers were inserted in Figures 2 and 3, according to your suggestion. In Figure 2, the percentage of the monthly growth rate of COVID-19 cases is presented by fixing the month of first symptoms and the group (with or without CVD). For example, in the CVD group, the growth rate of COVID-19 cases in April 2020 was 646,15% with respect to the month of the first symptoms immediately preceding. Note that, according to the absolute frequency, fixed the CVD group, 26 cases of COVID-19 occur in March 2020 and 194 cases in April 2020. To explain better the monthly growth rate of COVID-19, we add the following sentence in section Results:

“It is important to note that the growth rate of COVID-19 cases for a specific month of the first symptoms is calculated with respect to the month immediately preceding and the fixed group (CVD or non-CVD).”

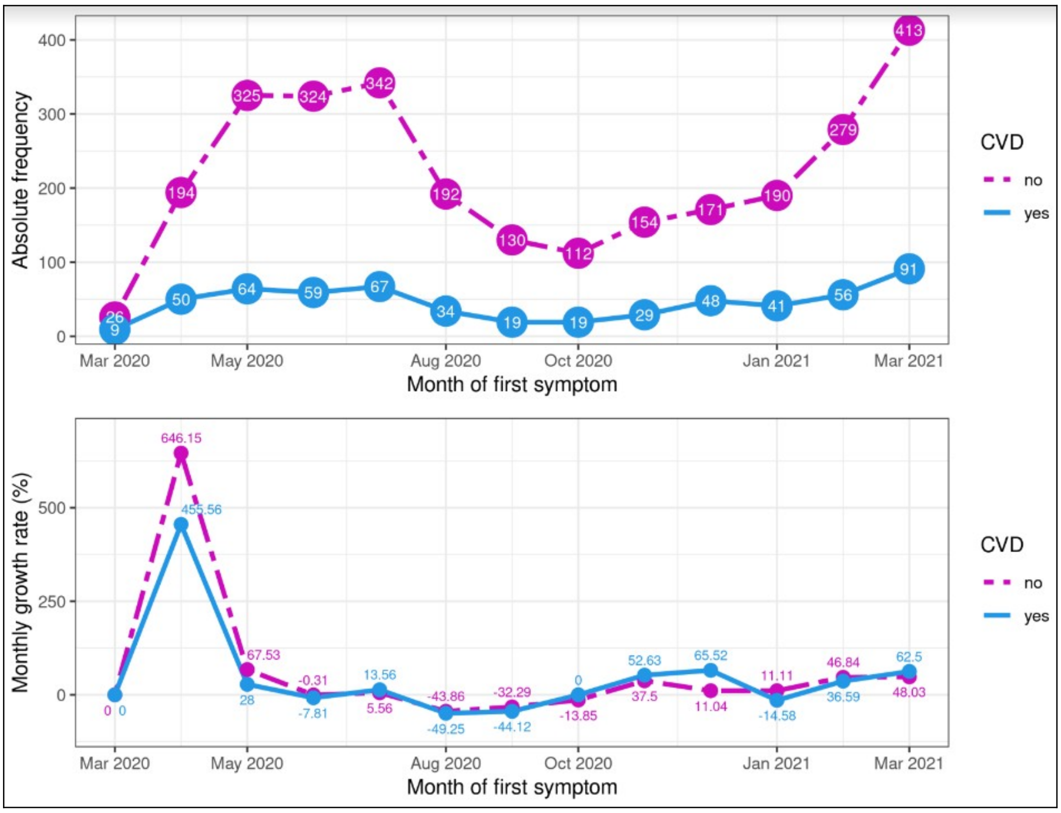

Figure 2. Absolute frequency and percentage of monthly growth rate of COVID-19 cases according to the month of the first symptom in the CVD and non-CVD groups.

In Figure 3, considering the group (CVD or non-CVD) and the month of first symptom fixed, the *a/b* notation represents the occurrence of *a* deaths among the total of dead and cured in the month (*b*). For example, in March 2020, the proportion of deaths in the CVD group was 55.56%, and the value 5/9 indicates that of the 9 individuals with first symptoms in this month, 5 died. We add the following sentence in the section Results to explain the notation in Figure 3:

"In Figure 3, considering the group (CVD or non-CVD) and the month of first symptom fixed, the notation *a/ b* indicates that of the *b* individuals with the first symptoms in a specific month, *a* died."

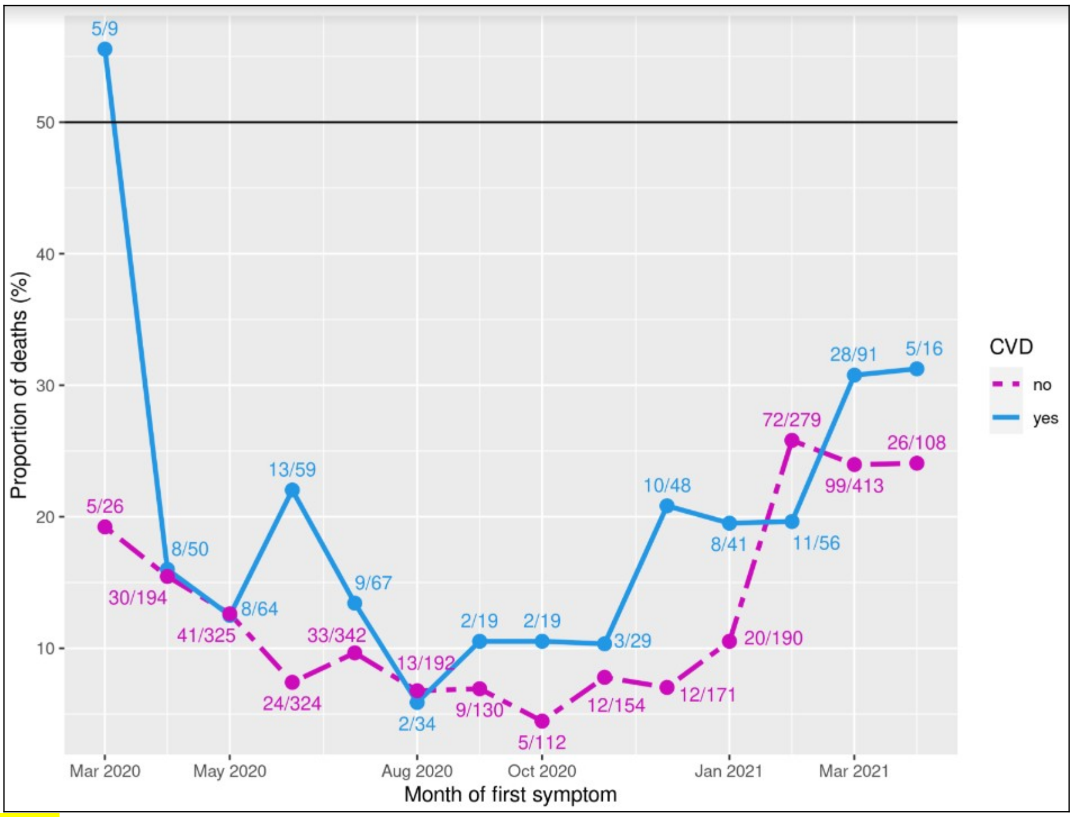

Figure 3: Percentage of mortality among patients with CVD, fixed the month of first symptom.

5.4. "This was different from what happened in the 2nd wave in which CVD patients were mostly affected." – How did you conclude this? Did you calculate the proportion out of the entire "maternal" population (including non-COVID)?

Response: All statements made in the text are related to the investigated group given by only confirmed and hospitalized cases of COVID-19 in pregnant and postpartum women aged 10 to 55 years who had in their notification the information about the presence or absence of CVD. We tried to clarify this sentence including a new analysis based on the results presented in the second graph of Figure 2, which is the percentage of the monthly growth rate of COVID-19 cases according to the month of the first symptom in the CVD and non-CVD groups. For this, we add the following sentence:

"Note that, if we consider the first wave as the period from March to October 2020, the average growth rate of COVID-19 cases calculated using geometric mean was 11,27% for the CVD group and 23,20% for the non-CVD group. In the second wave, from October 2020 to April 2021, the average growth rate of COVID-19 cases was 36,79% and 29,82% for the CVD and non-CVD groups, respectively. These numbers show us that in the 2nd wave, the virus was highly transmissible in both groups (CVD and non-CVD) in comparison with the first wave and, in particular, more aggressive in the CVD patients since they had a higher rate of contamination."

***5.5.* In table 1- What is included under "Hemopathy"? "Hepatopathy"? "Pneumopathy"? "Immunosuppression"? "Nephropathy"? How did you define obesity?**

Response: The comorbidities listed in Table 1 are the comorbidities that were mentioned in the case notification form, according to the Ministry of Health's data dictionary, referring only to chronic diseases prior to the reported condition. There is no information available on ICD codes. Obesity is considered when the body mass index is greater than 30.

***5.6.* What was the absolute mortality rate in every group?**

Response: The absolute number of deaths was 114 in the CVD group and 401 in the non-CVD group. This information is available in Table 4 and was inserted in Figure 3, stratified by month of first symptoms.

6. Conclusions - Most of the conclusions cannot be referred to without the above information. That is why, in fact, the paper is rejected.

Response: We are grateful for your suggestions and those of the other reviewers and we hope to have been able to clarify all doubts, allowing the acceptance of the study for publication.

***7.* Limitation - "limitations of the study, we should consider that the completion of the notification system included information about the presence or absence of CVD in 39.58% of the notifications, with a high percentage of loss." Can you clarify this sentence? Is approximately 60% of the patients' background status missing?**

Response: Thanks for your comment. We agree that there is a large proportion of missing data, which was even pointed out as one of the weaknesses of the study, however the loss of data in this study was lower than in other similar studies such as the one by the CDC where only 5.8% (7162) of 122653 cases reported had information about health conditions.

It is worth mentioning that we only use cases with the information called "presence" or "absence" of the disease, that is, we do not use it if the field is not filled in.

With this, we hope to minimize the bias of the study by avoiding the misinterpretation that the data not provided means the absence of disease.
